# Rural physical activity interventions in the United States: a systematic review and RE-AIM evaluation

**DOI:** 10.1186/s12966-019-0903-5

**Published:** 2019-12-27

**Authors:** Nishat Bhuiyan, Pritika Singh, Samantha M. Harden, Scherezade K. Mama

**Affiliations:** 1 Department of Kinesiology, The Pennsylvania State University, 23B Recreation Building, University Park, PA 16802 USA; 2 Department of Kinesiology, The Pennsylvania State University, 23B Recreation Building, University Park, PA 16802 USA; 3 Department of Human Nutrition, Foods, and Exercise, Virginia Tech, Blacksburg, VA 24060 USA; 4 Department of Kinesiology, The Pennsylvania State University, 268J Recreation Building, University Park, PA 16802 USA

**Keywords:** Physical activity, External validity, Internal validity, Rural health

## Abstract

**Background:**

Previous reviews of rural physical activity interventions were focused on intervention effectiveness and had reported overall mixed findings**.** The purpose of this systematic review was to apply the Reach, Efficacy, Adoption, Implementation and Maintenance (RE-AIM) framework to evaluate the extent to which rural physical activity interventions in the U.S. have reported on dimensions of internal and external validity and to offer suggestions for future physical activity interventions for rural U.S. populations.

**Methods:**

Pubmed, PsychINFO, CINAHL, PAIS, and Web of Science were searched through February 2019 to identify physical activity intervention studies conducted in rural regions in the U.S. with adult populations. Titles, abstracts, and full texts of articles were reviewed against inclusion and exclusion criteria. Data extraction from included articles included a summary of study details, rural classification system used, and the presence or absence of a total 61 RE-AIM indicators, including reach (*n* = 13), efficacy/effectiveness (*n* = 10), adoption (*n* = 21), implementation (*n* = 9), and maintenance (*n* = 8).

**Results:**

A total of 40 full-text articles representing 29 unique studies were included. Classifications of rurality included self-statements by authors (*n* = 19, 65.5%), population/census-based definitions (*n* = 3, 10.3%), Rural Urban Continuum Codes (*n* = 3, 10.3%), Rural Urban Commuting Area codes (*n* = 2, 6.9%), the 2014 Alabama Rural Health Association classification system (*n* = 1, 3.4%) and the U.S. Office of Management and Budget classification system (*n* = 1, 3.4%). Individual studies reported between 14.8 to 52.5% of total RE-AIM indicators. Studies reported 15.4 to 84.6% indicators for reach; 20.0 to 70.0% indicators for efficacy/effectiveness; 4.8 to 47.6% indicators for adoption; 11.1 to 88.9% indicators for implementation; and 0 to 25.0% indicators for maintenance.

**Conclusions:**

We found an overall poor reporting of components related to external validity, which hinders the generalizability of intervention findings, and a lack of consistency in the definition of rurality. Future research should focus on balancing factors of internal and external validity, and should aim to develop a greater understanding of how rurality influences health and behavior to provide contextual knowledge needed to advance the translation of physical activity interventions into practice in rural communities and reduce rural health disparities.

**Trial registration:**

The review protocol was registered with PROSPERO: CRD42019116308.

## Background

Rural areas cover about 97% of land in the United States (U.S.) and include about 20% of the population, or around 60 million residents [1]. Rural residents in the U.S. are less physically active than urban residents [2]. As a result, rural residents face increased rates of mortality and disease related to inactivity, such as obesity and heart disease, when compared to urban counterparts [3]. Adopting healthful behaviors, such as physical activity, reduces the risk of morbidity and mortality [4]. Thus, physical activity promotion among rural adults may have a major public health impact and help to reduce rural health disparities.

Several reviews have assessed the effectiveness of physical activity interventions among rural populations and have reported mixed findings [5–8]. Results of these reviews conclude that to be effective, interventions should include low- to moderate-intensity aerobic exercise [7], be personalized and tailored with multiple intervention contacts [5], and incorporate behavior change theory [8]. Among previous reviews of rural physical activity interventions [5–8], only one review by Cleland and colleagues included a meta-analysis, which demonstrated no overall effect of interventions on physical activity [6]. This meta-analysis also demonstrated an intervention effect in favor of studies using objective measures of physical activity, but no intervention effect among studies using self-report measures of physical activity [6].

Although the literature shows there are promising components to existing physical activity interventions for rural residents, previous reviews have cited mixed findings and high risk of bias among included studies as major limitations, and have thus been unable to draw strong conclusions regarding the effectiveness of physical activity interventions in rural settings [5–8]. Furthermore, persistent disparities in physical activity between rural and urban residents suggest that effective interventions have not yet been effectively translated and implemented within rural communities to improve population health. The translation of interventions into practice is challenging, especially within complex environments with limited resources, such as rural communities [9]. Rural communities are characterized by multifaceted physical and social features; for example, in addition to unique geographic features of rural environments and longer distances to reach health services, there are also higher rates of unemployment, uninsured, and poverty among rural residents [10, 11]. To successfully translate evidence-based interventions into these complex rural communities, it is critical to examine the internal and external validity of interventions [12, 13].

The RE-AIM framework can be applied to evaluate the internal and external validity of interventions [14, 15]. The purpose of the framework is to help guide the dissemination and implementation of evidence-based interventions into practice [14, 16]. Specifically, the RE-AIM framework assesses the dimensions of reach, efficacy/effectiveness, adoption, implementation, and maintenance in order to determine the public health impact of interventions [14]. The dimensions of reach, which reflects the number, proportion, and representativeness of intervention participants, adoption, which reflects the number, proportion, and representativeness of intervention settings and staff, and maintenance, which at the setting level reflects if an intervention integrates into routine organizational practices and policies, allow researchers to evaluate external validity [14, 16]. The dimensions of efficacy/effectiveness, which reflects impact of an intervention on important outcomes, and implementation, which reflects intervention participants and staff fidelity to an intervention’s protocol, allow researchers to evaluate internal validity [14, 16]. RE-AIM has been used to assess the internal and external validity of physical activity interventions and to provide recommendations for future work in diverse populations, including breast cancer survivors, family caregivers, and Latin American populations [17–19]. For example, when the RE-AIM framework was applied to examine physical activity interventions in breast cancer survivors, White and colleagues demonstrated that while a majority of studies reported dimensions reflecting internal validity, dimensions reflecting external validity were rarely reported, thus limiting generalizability of study findings [17].

A comprehensive review of the internal and external validity of physical activity interventions in rural populations is currently lacking. This gap in the literature is coupled with a lack of certainty regarding the effectiveness of physical activity interventions in rural populations, which may be due to the poor quality of studies and high risk of bias, which limits the ability to draw strong conclusions as shown in Cai and colleagues’ review [5]. This lack of certainty may also be due to differences in rurality across countries in previous reviews, or other contextual factors that have not previously been explored (e.g., intervention delivery staff, setting) [6, 7]. Thus, the purpose of this review is to 1) evaluate the extent to which physical activity interventions in rural populations in the U.S. have reported on dimensions of internal and external validity using the RE-AIM framework, and 2) offer suggestions on the design and reporting of future physical activity interventions for rural U.S. populations to enhance their ability to be widely implemented and disseminated to improve population health. Since there is no single widely accepted rural classification system [20], we restricted our review to only studies conducted in the U.S. and summarized the different measures of rurality used by study authors to aid the generalizability of findings.

## Methods

### Protocol and registration

This systematic review is registered with the PROSPERO international prospective register of systematic reviews (registration number CRD42019116308) at the Centre for Reviews and Dissemination, University of York, UK, and adheres to the Preferred Reporting Items for Systematic Reviews and Meta-Analyses (PRISMA) reporting guidelines [21]. The PRISMA checklist is available as Additional file 1**.**

### Eligibility criteria

Study inclusion criteria are described in Table 1. Articles were excluded if they: 1) were not conducted in the U.S., 2) were not an intervention study, 3) did not include an adult population (18+ years old, or mean age < 65 years), consistent with previous reviews [6], and 4) did not report pre- and post-intervention measures of physical activity, exercise, or fitness as an intervention outcome, consistent with previous reviews [7]. Furthermore, while the goal of this review is to apply the RE-AIM framework to evaluate rural physical activity interventions, explicitly stating that RE-AIM indicators were used for reporting was not part of the eligibility criteria when searching for studies.

**Table 1:** Study inclusion criteria

Data type	Inclusion criteria
Participants	Adults (18+ years old, or mean age < 65 years) residing in rural areas in the U.S. as described by study authors
Language	English
Study design	Randomized controlled trials and non-randomized trials with a control group (including quasi-experimental and natural experiment studies)
Control condition	Any comparator: active control, inactive control, or participants as their own control (i.e., pre- and post-measures)
Intervention	Increasing physical activity, exercise, or fitness in a rural setting is a goal of the intervention
Measurement	Assesses physical activity/exercise/fitness among participants at baseline and post-intervention
Outcome	Physical activity
	Exercise
	Fitness

### Search strategy

The following five electronic databases were searched for articles: Pubmed (January 1996–February 10, 2019), PsychINFO (1887-February 10, 2019), CINAHL (1961-February 10, 2019), PAIS (1972-February 10, 2019), and Web of Science (1900-February 10, 2019). The search was limited to original research articles published in English from each database’s inception through October 9, 2018 and updated on February 10, 2019. The search strategy was developed in consultation with a health sciences librarian and included the following search concepts: 1) rural population, rural health services, or rural health; 2) exercise, physical activity, walking, jogging, bicycling, or recreation; and 3) intervention studies, health promotion, or wellness programs (full search strategy is available as Additional file 2). The reference lists of all included full-text articles were further hand searched to identify any additional articles meeting the inclusion criteria, or any companion articles. A companion article is any article related to the primary study that may include additional intervention details. For example, some studies publish study protocols separately from the primary outcomes, in which additional RE-AIM indicators are reported.

### Study selection

Search results were managed using EndNote X9 reference manager software (Clarivate Analytics, Philadelphia, PA). Citation details for all articles (e.g., year of publication, authors, journal name, title, abstract) were downloaded and loaded into a single file. Duplicate articles were identified using Endnote X9, reviewed, and removed from the database. Two coders (NB and PS) independently completed initial screening of titles and abstracts, separately. The full texts of the remaining articles were then independently reviewed against inclusion and exclusion criteria by two coders (NB and PS). The inter-rater reliability, which was calculated using Cohen’s κ, was .82, indicating a high degree of agreement [22]. Disagreements between coders were discussed until consensus was reached. Reasons for exclusion were documented at the full text screening stage.

### Data extraction and analysis/synthesis

A coding tool adapted from a previous systematic review using the RE-AIM framework [23] was used by two coders (NB and PS) to independently extract and code data from included articles. Disagreements between coders regarding extracted data were discussed until consensus was reached. Extracted data included citation details, companion article citation details, definition and classification of rurality, intervention outcome (e.g., physical activity, exercise, or fitness), target population, study setting, and study design. For each of the five RE-AIM dimensions, the presence or absence of indicators were coded (yes/no), and if present, a description of the indicator was extracted. A total of 61 RE-AIM indicators were coded, including indicators to describe reach (*n* = 13), efficacy/effectiveness (*n* = 10), adoption (*n* = 21), implementation (*n* = 9), and maintenance (*n* = 8), which are described in Table 2. Data synthesis included a narrative description of primary studies and frequency counts and percentages across reported RE-AIM indicators.

**Table 2:** Inclusion of RE-AIM indicators across all studies

RE-AIM Dimension	RE-AIM Indicators	N (%)
Reach	Described target population	29 (100.0)
	Demographic & behavioral information	23 (79.3)
	Method to identify target population	23 (79.3)
	Recruitment Strategies	17 (58.6)
	Inclusion criteria	25 (86.2)
	Exclusion criteria	24 (82.7)
	Number eligible and invited (exposed) to recruitment	18 (62.1)
	Sample size	29 (100.0)
	Participation rate	18 (62.2)
	Demographic comparisons between sample and population	4 (13.8)
	Statistically significant comparisons between sample and population	3 (10.3)
	Cost of recruitment	0 (0.0)
	Use of qualitative methods to measure reach	0 (0.0)
Effectiveness/Efficacy	Results at program completion	29 (100.0)
	Report of Mediators	3 (10.3)
	Report of Moderators	3 (10.3)
	Intent-to-treat or present at follow-up	14 (48.3)
	Imputation procedures	5 (17.2)
	Quality of life measure	8 (27.6)
	Measure of unintended consequences (negative)	1 (3.4)
	Percent attrition at program completion	24 (82.7)
	Cost effectiveness	0 (0.0)
	Use of qualitative methods to measure efficacy/effectiveness	8 (27.6)
Adoption - Setting level	Number eligible and invited (exposed) sites	9 (31.0)
	Number of participating sites	9 (31.0)
	Participation rate	18 (62.1)
	Description of targeted location	23 (79.3)
	Inclusion/exclusion criteria of setting	11 (37.9)
	Description of intervention location	17 (58.6)
	Method to identify setting	8 (27.6)
	Demographic comparisons between site and target site	3 (10.3)
	Statistically significant comparisons between site and target site	0 (0.0)
	Average number of persons served per setting	4 (44.4)
Adoption – Staff level	Number eligible and invited (exposed) staff	0 (0.0)
	Number participating in delivery	9 (31.0)
	Participation rate of staff	3 (10.3)
	Method to identify target delivery agent	11 (37.9)
	Level of expertise of delivery agent	16 (55.2)
	Inclusion/exclusion criteria of delivery agent	5 (17.2)
	Demographic comparisons between staff and target staff	1 (3.4)
	Statistically significant comparisons between site and target staff	0 (0.0)
	Measures of cost of adoption	3 (10.3)
	Dissemination beyond originally planned	0 (0.0)
	Use of qualitative methods to measure adoption	1 (3.4)
Implementation	Theories	21 (72.4)
	Intervention number of contacts	25 (86.2)
	Timing of contacts	24 (82.8)
	Duration of contacts	12 (41.4)
	Extent protocol delivered as intended	5 (17.2)
	Consistency of implementation across setting and delivery agents	5 (17.2)
	Participant attendance/completion rates	13 (44.8)
	Measure of cost	3 (10.3)
	Use of qualitative methods to measure implementation	3 (10.3)
Maintenance - Individual	Individual behavior assessed at some duration following the completion of the intervention	4 (13.8)
	Attrition at follow-up	4 (13.8)
	Use of qualitative methods to measure individual maintenance	0 (0.0)
Maintenance - Organizational	Report alignment to organization mission	1 (3.4)
	Is the program still in place?	0 (0.0)
	Was the program institutionalized?	0 (0.0)
	Site attrition at follow-up	0 (0.0)
	Use of qualitative methods to measure organizational level maintenance	0 (0.0)

## Results

**Fig. 1:**
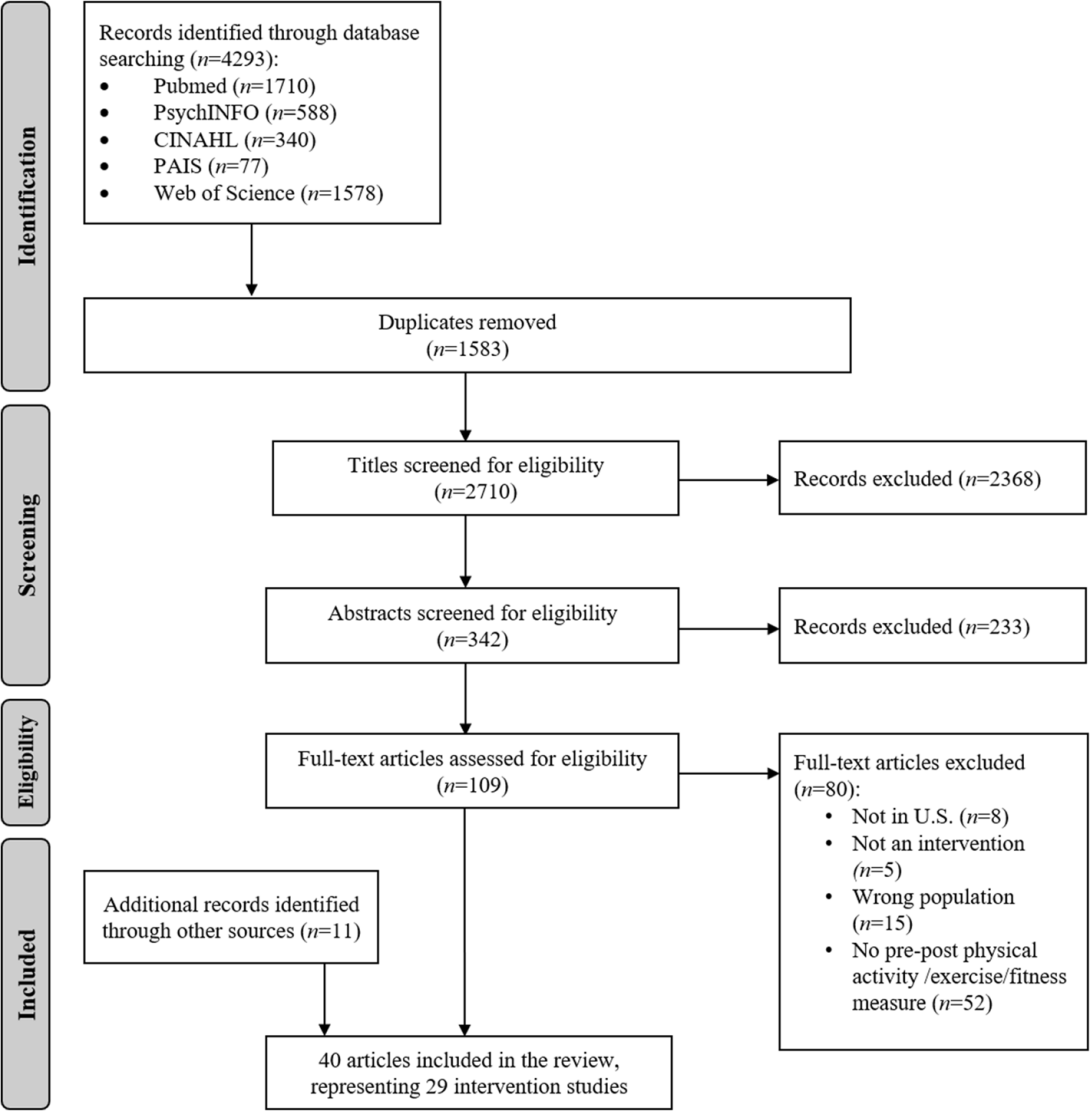
Summary of articles identified, excluded and included in the systematic review

Our search yielded 2710 articles after the exclusion of duplicates (Fig. 1). Of those, 2601 articles were excluded during title and abstract screening, yielding 109 articles for full-text review. An additional 80 articles were excluded after review of the full text, and 11 additional articles were identified from hand searching the reference lists of included articles, resulting in a total of 40 full-text articles representing 29 unique studies [24–63].

### Study and participant characteristics

Studies included in this review are described in Additional file 3. Overall, study sample sizes ranged from 15 to 1257 (*M* = 217.8, *SD* = 263.2), and 51.7% (*n* = 15) of studies included exclusively women. Studies ranged in duration from 5 weeks to 96 weeks (*M* = 31.7, *SD* = 25.1). In-person intervention settings included churches (*n* = 6, 20.7%), a mix of various community locations (*n* = 5, 17.2%), worksites (*n* = 3, 10.3%), medical practices/clinics (*n* = 1, 3.4%), participants’ homes, (*n* = 1, 3.4%), community or recreation centers (*n* = 1, 3.4%), or were unreported (*n* = 2, 6.9%). Out of the 10 interventions that were not delivered in person, a total of 6 interventions (20.7%) used telephone-based delivery, 2 interventions (6.9%) were website-based, and 2 interventions (6.9%) were SMS message-based. Classifications of rurality included self-statements by authors (with no description of a standardized classification system) (*n* = 19, 65.5%), population/census-based definitions (*n* = 3, 10.3%), Rural Urban Continuum Codes (RUCC) (n = 3, 10.3%), and Rural Urban Commuting Area (RUCA) codes (*n* = 2, 6.9%). Additionally, one study used the Alabama Rural Health Association 2014 classification system to define six counties in Alabama as rural [54], and one study used the U.S. Office of Management and Budget (OMB) classification system to define Delaware County, NY, as rural [52]. Most (69.0%, *n* = 20) studies reported a significant improvement in at least one physical activity, exercise, or fitness outcome, and 9 (31.0%) interventions reported no significant improvements in any physical activity, exercise, or fitness outcome.

### RE-AIM indicators

No studies explicitly stated that RE-AIM indicators were used for reporting. Overall, individual studies reported 9 to 32 (Median = 20) out of a total of 61 (14.8 to 52.5%; Median = 32.8%) RE-AIM indicators. Studies reported 2–11 (Median = 7) indicators out of 13 indicators (15.4 to 84.6%; Median = 53.8%) for reach; 2–7 (Median = 3) out of 10 indicators (20.0 to 70.0%; Median = 30.0%) for efficacy/effectiveness; 1–10 (Median = 5) out of 21 indicators (4.8 to 47.6%, Median = 23.8%) for adoption; 1–8 (Median = 4) out of 9 indicators (11.1% to 88.%; Median = 44.4%) for implementation; and 0–2 (Median = 0) out of 8 indicators (0 to 25.0%; Median = 0.0%) for maintenance. The number of indicators reported by each included article is shown in Table 3, and the total number and percentage of studies reporting on each RE-AIM indicator is shown in Table 2**.**

**Table 3:** Number of indicators of each RE-AIM dimension across all articles (*N* = 29)

Author, year	Reach (*n* = 13)	Effectiveness/Efficacy (*n* = 10)	Adoption (*n* = 21)	Implementation (*n* = 9)	Maintenance (*n =* 8)	Total (*N* = 61)
	[N (%)]	[N (%)]	[N (%)]	[N (%)]	[N (%)]	[N (%)]
Anson & Madras, 2016 [24]	4 (30.8)	3 (30.0)	1 (4.8)	2 (22.2)	0 (0.0)	10 (16.4)
Befort et al., 2010 [25]	9 (69.2)	6 (60.0)	8 (38.1)	8 (88.8)	0 (0.0)	31 (50.)
Befort et al., 2012 [26]	10 (76.9)	5 (50.0)	5 (23.8)	6 (66.7)	0 (0.0)	26 (42.6)
Benson et al., 2019 [27]	8 (61.5)	4 (40.0)	4 (19.0)	5 (55.6)	0 (0.0)	21 (34.4)
Campbell et al., 2002 [28]	10 (76.9)	3 (30.0)	10 (47.6)	2 (22.2)	0 (0.0)	25 (41.0)
Campbell et al., 2004 [31]	9 (69.2)	2 (20.0)	9 (42.9)	3 (33.3)	0 (0.0)	23 (37.7)
Campbell et al., 2012 [32]	6 (46.2)	5 (50.0)	6 (28.6)	3 (33.3)	0 (0.0)	20 (32.8)
Ely et al., 2008 [34]	7 (53.8)	4 (40.0)	7 (33.3)	5 (55.6)	0 (0.0)	23 (37.7)
Fahs et al., 2013 [36]	7 (53.8)	3 (30.0)	6 (28.6)	4 (44.4)	0 (0.0)	20 (32.8)
Farag et al., 2010 [37]	5 (38.5)	2 (20.0)	7 (33.3)	3 (33.3)	0 (0.0)	17 (27.9)
Fazzino et al., 2017 [38]	2 (15.4)	4 (40.0)	1 (4.8)	2 (22.2)	0 (0.0)	9 (14.8)
Folta et al., 2009 [41]	11 (84.6)	2 (20.0)	7 (33.3)	6 (66.7)	0 (0.0)	26 (42.6)
Gore et al., 2019 [42]	9 (69.2)	4 (40.0)	5 (23.8)	4 (44.4)	0 (0.0)	22 (36.1)
Greaney et al., 2017 [43]	6 (46.1)	2 (20.0)	2 (20.0)	2 (22.2)	0 (0.0)	12 (19.7)
Griffin et al., 2018 [45]	7 (53.8)	2 (20.0)	1 (4.8)	3 (33.3)	0 (0.0)	13 (19.7)
Hageman et al., 2014 [46]	10 (76.9)	3 (30.0)	3 (14.3)	5 (55.6)	2 (25.0)	23 (37.7)
Hu et al., 2014 [47]	8 (61.5)	2 (20.0)	4 (19.0)	3 (33.3)	2 (25.0)	19 (31.1)
Keyserling et al., 2016 [48]	5 (38.5)	2 (20.0)	2 (9.5)	2 (22.2)	0 (0.0)	11 (18.0)
Kim et al., 2008 [50]	9 (69.2)	3 (30.0)	9 (42.9)	4 (44.4)	0 (0.0)	25 (41.0)
Lilly et al., 2014 [51]	4 (30.8)	3 (30.0)	4 (19.0)	8 (88.8)	0 (0.0)	19 (31.1)
Marigliano et al., 2016 [52]	7 (53.8)	2 (20.0)	3 14.3)	1 (11.1)	0 (0.0)	13 (21.3)
Parker et al., 2010 [53]	11 (84.6)	4 (40.0)	10 (47.6)	7 (77.8)	0 (0.0)	32 (52.5)
Scarinci et al., 2014 [54]	10 (76.9)	4 (40.0)	8 (38.1)	6 (66.7)	2 (25.0)	30 (49.2)
Spurrier et al., 2018 [55]	6 (46.2)	2 (40.0)	4 (19.0)	4 (44.4)	0 (0.0)	16 (26.2)
Thomson et al., 2016 [56]	5 (38.5)	2 (20.0)	2 (9.5)	4 (44.4)	0 (0.0)	13 (21.3)
Tussing-Humphreys et al., 2013 [58]	8 (61.5)	2 (20.0)	7 (33.3)	4 (44.4)	0 (0.0)	21 (34.4)
Warren et al., 2010 [60]	5 (38.5)	2 (20.0)	4 (19.0)	1 (11.1)	0 (0.0)	12 (19.4)
Wilcox et al., 2013 [61]	6 (46.2)	3 (30.0)	5 (23.8)	1 (11.1)	0 (0.0)	15 (24.6)
Zoellner et al., 2013 [63]	7 (53.8)	7 (70.0)	5 (23.8)	6 (66.7)	1 (12.5)	26 (42.6)

### Single studies vs. multiple papers

Compared to the number of RE-AIM indicators reported by individual studies (*n* = 29), when companion articles (*n* = 10) were included in the synthesis, studies (*N* = 40) reported 10 to 39 (16.4 to 63.9%) out of a total of 61 RE-AIM indicators. Studies reported 3–12 indicators (23.1 to 92.3%) out of 13 indicators for reach; 3–9 indicators (30.0 to 90.0%) out of 10 indicators for efficacy/effectiveness; 2–15 indicators (9.5 to 71.4%) out of 21 indicators for adoption; 1–7 indicators (11.1 to 77.8%) out of 9 indicators for implementation; and 0–4 indicators (12.5 to 50.0%) out of 8 indicators for maintenance.

## Discussion

Previous reviews of physical activity interventions in rural populations identified features showing promise for intervention effectiveness but had reported overall mixed findings [5–8]. The current study extended this literature by examining the extent to which physical activity interventions in rural populations reported on reach, efficacy and effectiveness, adoption, implementation, and maintenance and provides recommendations for future research based on findings. We found in addition to the high risk of bias and poor quality of studies cited previously [5–8], there is an overall low reporting of RE-AIM dimensions, particularly in adoption and maintenance, which are dimensions related to external validity. Low reporting of dimensions related to external validity may mean that research aimed at rural physical activity and health promotion is currently not placing enough emphasis on improving factors such as the number, proportion and representativeness of settings and staff members agreeing to initiate an intervention, and sustained intervention delivery at the setting or staff level. This may be negatively impacting efforts to translate evidence-based physical activity interventions into rural communities in the U.S. and hindering the widespread dissemination of these interventions, contributing to the persistence of rural health disparities.

We found many similarities when comparing our results to previous RE-AIM reviews of physical activity interventions in other populations, including Latin Americans, Canadian and U.S. family caregivers, and breast cancer survivors [17–19]. Similar to the current study, previous reviews found that articles reported more frequently on reach, efficacy/effectiveness, and implementation and less frequently on adoption and maintenance [17–19]. The lack of reporting on factors related to external validity is an issue consistently seen among physical activity interventions in diverse populations. This lack of information about external validity, which provides critical knowledge about whether interventions can be effective in other settings and populations or with other staffing and resources, obstructs the translation of research into public health practice [13]. Thus, it is imperative that future physical activity intervention studies more accurately report across all RE-AIM dimensions, and additional focus is needed on reporting on factors related to external validity, such as characteristics related to intervention delivery agents and intervention sites.

We found there was limited reporting on measures of unintended and negative consequences and on mediators and moderators. Measuring unintended and negative consequences allows for researchers to determine whether an otherwise effective intervention may have unanticipated consequences and may cause unintended harm. Measuring moderator variables allow researchers to determine characteristics that influence the direction and strength of the relationship between the intervention and outcome, which can then be used to identify subgroups with greater or lesser likelihood to respond favorably to an intervention. Measuring mediators allows researchers to identify variables that explain the extent to which that variable accounts for the relationship between the intervention and outcome, and could reflect the underlying mechanisms of the intervention. These are all critical factors related to intervention effectiveness, and we therefore encourage researchers to include these measures in addition to assessing primary intervention outcomes.

Additionally, there was limited reporting related to cost. None of the included studies in the current review reported on costs of recruitment or cost of intervention adoption, and only 10.3 and 3.4% reported the cost of intervention implementation and cost-effectiveness, respectively. Thus, there is limited evidence for the cost-effectiveness of physical activity interventions in rural communities, which impacts practice and policy [26]. Existing reviews on the cost-effectiveness of physical activity interventions have been mixed, and there are few interventions found to be cost-effective [26]. Policy makers require the appraisal of the cost and benefits of public health programs to inform decisions about funding and resource allocation, which makes information on intervention cost-effectiveness critical for making public health decisions on physical activity promotion [64–66]. The existing limited economic research in the field of physical activity promotion in rural areas implies that current investments into this area may be based on assumptions, rather than the effectiveness and cost of specific interventions. Thus, researchers should be encouraged to increase reporting related to cost and to identify strategies for improving intervention cost-effectiveness, in order to contribute to the evidence-base from which to draw insights on the cost-effectiveness of rural behavioral health interventions and to inform policy and practice.

Additionally, we also found that when companion articles were included in data extraction and synthesis, studies reported more RE-AIM indicators compared to individual studies, similar to previous reviews [23]. We therefore agree with previous recommendations that authors report dimensions across multiple companion articles [17, 23], because this may alleviate concerns about journal space and manuscript length restrictions and may facilitate more balanced and thorough reporting of RE-AIM dimensions. Furthermore, given that no studies explicitly stated the use of RE-AIM indicators for study evaluation, we encourage future researchers to use the RE-AIM framework for both intervention planning and evaluation. This would allow for future evaluations of fidelity to the RE-AIM framework in the field of physical activity promotion in rural communities.

In addition to our RE-AIM findings, we noted the different classification systems used to define rurality in studies, which included population/census-based definitions, Rural Urban Continuum Codes (RUCC), and Rural Urban Commuting Area (RUCA) codes [20]. Most included studies simply stated their population or setting was rural, and few interventions used the same classification system to define rurality. A previous review by Cleland and colleagues of rural physical activity interventions in multiple countries noted differences in the rural classification systems used in studies [6]. This study demonstrates that even when assessing physical activity intervention research exclusively in the U.S., discrepancies remain in the operational definitions used to categorize rural settings and populations. This is an issue because research findings based on inconsistent definitions of rural may appear to conflict and can result in considerably different conclusions and policy implications [20].

However, we do not suggest that the solution moving forward is to select one of the existing definitions of rurality as the standard rurality classification to be used among public health researchers. Existing common definitions of rurality are based on factors such as population size, density, proximity, degree of urbanization, adjacency and relationship to a metropolitan area, principal economic activity, economic and trade relationships, and work commutes [20]. These definitions do not include key factors, such as sociodemographic characteristics, environmental characteristics, and healthcare and resource availability, which may be important indicators for identifying populations at risk for negative health behaviors and outcomes [67]. Thus, we encourage that researchers further explore these factors in rural areas in order to develop a greater understanding of how rurality influences residents’ health and behavior. A greater understanding of what it means to be rural and how that influences health and behavior, and applying that understanding in selecting target intervention samples and settings, would provide greater context to physical activity interventions in rural settings. This contextual knowledge could then allow researchers to make comparisons across studies despite the lack of a standardized rural classification system, which is critical for facilitating the translation of interventions across rural settings and populations.

The current review makes a unique contribution to the literature on the examination of the internal and external validity of physical activity interventions in rural adults in the U.S. In a previous paper, Umstattd Meyer and colleagues (2016) assessed and outlined gaps in the evidence-base for an ecological model of active living for rural populations [68]. While Umstattd Meyer and colleagues (2016) provided a broad overview of the literature on the multi-level influences on active living in rural communities, including cross-sectional studies of determinants and correlates of physical activity [68], the current review extended this work by focusing on and systematically reviewing the internal and external validity of physical activity intervention studies in rural communities.

Additional strengths of this current review include an exhaustive search strategy, developed and conducted in consultation with a trained librarian, and well-defined enumeration of inclusion and exclusion criteria. Despite study strengths, there are limitations that should be noted. First, inclusion criteria were restricted to articles published in English and studies conducted in the U.S., and interventions targeting older adults (or study samples with a mean age > 65 years old) were excluded. Inclusion and exclusion criteria were kept similar to a previous review to aid comparisons [6]. However, the national physical activity guidelines are identical for adults and older adults [69], and future reviews should incorporate interventions targeting older adults for a more inclusive assessment of physical activity interventions in rural settings. Secondly, we summarized reporting across dimensions related to both internal and external validity. We did not focus on the effectiveness of physical activity interventions in rural populations, which has been reported previously but warrants further study [6]. Furthermore, due to the multiple reporting and evaluation tools available, a limitation applicable to many RE-AIM studies is the lack of consensus regarding a specific tool [70]. Previous RE-AIM reviews used a smaller number of total RE-AIM indicators [18, 71], making it difficult to make a direct comparison to the current study results. However, the use of a data extraction tool with a larger number of indicators allowed us to gain a more comprehensive understanding of the reporting of internal and external validity in physical activity interventions in rural adults.

## Conclusions

In sum, this systematic review provides information relevant to physical activity promotion in rural populations in the U.S. The poor reporting of components related to external validity, such as adoption and maintenance, may be indicating that improving factors such as representativeness or sustained intervention at the setting and staff level are currently not prioritized among public health researchers. This may be contributing to the limited widespread dissemination of effective physical activity interventions among rural populations; therefore, we recommend that researchers focus on balancing factors of internal and external validity and reporting these dimensions rigorously. Furthermore, we encourage researchers to continue testing strategies for increasing physical activity among rural populations, given the finding that many included interventions did not improve physical activity. Lastly, while there may not be a universally-accepted or standardized definition of rurality, steps need to be taken in order to facilitate the comparison of studies across rural settings. Therefore, we encourage researchers to elucidate the concept of rurality by further exploring factors influencing the health and behavior of rural residents, and to use that contextual knowledge when selecting intervention participants and settings.

## Supplementary information

**Additional file 1.** PRISMA Checklist. Description: Completed PRISMA checklist.

**Additional file 2.** Database search strategies. Description: Full individual search strategies for Pubmed, PsychINFO, CINAHL, PAIS, and Web of Science.

**Additional file 3.** Characteristics of original studies included in review. Description: The citation details, sample characteristics, location/setting, rurality classification, and summary of findings of included articles.

## Data Availability

Not applicable.
