# Supplementary material for: Rural physical activity interventions in the United States: a systematic review and RE-AIM evaluation
Source: Int J Behav Nutr Phys Act. 2019 Dec 27;16:140. doi: 10.1186/s12966-019-0903-5 (PMC6935185; doi:10.1186/s12966-019-0903-5)
Supplement: Supplementary file 2 — Additional file 2. Database search strategies. Description: Full individual search strategies for Pubmed, PsychINFO, CINAHL, PAIS, and Web of Science. [file 12966_2019_903_MOESM2_ESM.docx]

**Additional File 2:** Database search strategies.

| **Search strategy for Pubmed** |
| --- |
| ("Rural Population"[Mesh] OR "Rural Population"[tiab] OR "Rural Populations"[tiab] OR "Rural Spatial Distribution"[tiab] OR “Rural Spatial Distributions”[tiab] OR "Rural Communities"[tiab] OR "Rural Community"[tiab] OR "Rural Health Services"[Mesh] OR "Rural Health Services"[tiab] OR “Rural Health Service”[tiab] OR "Rural Health Center"[tiab] OR "Rural Health Centers"[tiab] OR "Rural Health"[Mesh] OR “Rural Health”[tiab] OR "Suburban Population"[Mesh] OR "Suburban Population"[tiab] OR "Suburban Populations"[tiab] OR "Nonmetropolitan Population"[tiab] OR "Nonmetropolitan Populations"[tiab] OR "Suburbanization"[tiab] OR "Farms"[Mesh] OR "Farms"[tiab] OR "Farm"[tiab] OR "Farmland"[tiab] OR "Farmlands"[tiab] OR "Vineyard"[tiab] OR "Vineyards"[tiab] OR "rural"[tiab] OR "micropolitan"[tiab] OR "nonmetropolitan"[tiab] OR "non-metropolitan"[tiab] OR "nonmetro"[tiab] OR "non-metro"[tiab] OR "agricultural"[tiab] OR "countryside"[tiab] OR "small town"[tiab] OR "provincial"[tiab] OR "pastoral"[tiab] OR “rural environment”[tiab] OR “rural environments”[tiab] OR “suburban environment”[tiab] OR “suburban environments”[tiab] OR “suburban areas”[tiab] OR “suburban areas”[tiab] OR “suburban”[tiab] OR “suburban health”[tiab] OR “agriculture”[tiab] OR “rural area”[tiab] OR “rural areas”[tiab] OR “suburbs”[tiab] OR “nonmetropolitan area”[tiab] OR “nonmetropolitan areas”[tiab])  AND  (“Exercise”[Mesh] OR “Exercise”[tiab] OR “Exercises”[tiab] OR “Physical Activity”[tiab] OR “Physical Activities” [tiab] OR “Physical Exercise”[tiab] OR “Physical Exercises”[tiab] OR “Acute Exercise”[tiab] OR “Acute Exercises”[tiab] OR “Isometric Exercises”[tiab] OR “Isometric Exercise”[tiab] OR “Aerobic Exercise”[tiab] OR “Aerobic Exercises”[tiab] OR “Exercise Training”[tiab] OR “Exercise Trainings”[tiab] OR “Running”[Mesh] OR “Running”[tiab] OR “Runnings”[tiab] OR “Jogging”[Mesh] OR “Jogging”[tiab] OR “Joggings”[tiab] OR “Walking”[Mesh] OR “Walking” [tiab] OR “Ambulation”[tiab] OR “Physical Conditioning, Human”[Mesh] OR “Human Physical Conditioning”[tiab] OR “Human Physical Training”[tiab] OR “Circuit-Based Exercise”[Mesh] OR “Circuit-Based Exercise”[tiab] OR “Circuit Based Exercise”[tiab] OR “Circuit-Based Exercises”[tiab] OR “Circuit Based Exercises”[tiab] OR “Circuit Training”[tiab] OR “Circuit Trainings”[tiab] OR “High-Intensity Interval Training”[Mesh] OR “High-Intensity Interval Training”[tiab] OR “High Intensity Interval Training”[tiab] OR “High Intensity Interval Trainings”[tiab] OR “High-Intensity Interval Trainings”[tiab] OR “High-Intensity Intermittent Exercise”[tiab] OR “High-Intensity Intermittent Exercises”[tiab] OR “High Intensity Intermittent Exercise”[tiab] OR “High Intensity Intermittent Exercises”[tiab] OR “Sprint Interval Training”[tiab] OR “Sprint Interval Trainings”[tiab] OR “Plyometric Exercise”[Mesh] OR “Plyometric Exercise”[tiab] OR “Plyometric Exercises”[tiab] OR “Plyometric Drill”[tiab] OR “Plyometric Drills”[tiab] OR “Plyometric Training”[tiab] OR “Plyometric Trainings”[tiab] OR “Plyometrics”[tiab] OR “Stretch-Shortening Exercise”[tiab] OR “Stretch Shortening Exercise”[tiab] OR “Stretch Shortening Exercises”[tiab] OR “Stretch-Shortening Exercises”[tiab] OR “Stretch-Shortening Cycle Exercise”[tiab] OR “Stretch Shortening Cycle Exercise”[tiab] OR “Stretch Shortening Cycle Exercises”[tiab] OR “Stretch-Shortening Cycle Exercises”[tiab] OR “Stretch-Shortening Drill”[tiab] OR “Stretch Shortening Drill” [tiab] OR “Stretch Shortening Drills”[tiab] OR “Stretch-Shortening Drills”[tiab] OR “Resistance Training”[Mesh] OR “Resistance Training”[tiab] OR “Strength Training”[tiab] OR “Weight-Lifting Strengthening Program”[tiab] OR “Weight Lifting Strengthening Program”[tiab] OR “Weight Lifting Strengthening Programs”[tiab] OR “Weight-Lifting Strengthening Programs”[tiab] OR “Weight-Lifting Exercise Program”[tiab] OR “Weight Lifting Exercise Program”[tiab] OR “Weight Lifting Exercise Programs”[tiab] OR “Weight-Lifting Exercise Programs”[tiab] OR “Weight-Bearing Strengthening Program”[tiab] OR “Weight Bearing Strengthening Program”[tiab] OR “Weight Bearing Strengthening Programs”[tiab] OR “Weight-Bearing Strengthening Programs”[tiab] OR “Weight-Bearing Exercise Program”[tiab] OR “Weight Bearing Exercise Program”[tiab] OR “Weight Bearing Exercise Programs”[tiab] OR “Weight-Bearing Exercise Programs”[tiab] OR “Swimming”[Mesh] OR “Swimming”[tiab] OR “Weight Lifting”[Mesh] OR “Weight Lifting”[tiab] OR “Weight Liftings”[tiab] OR “Recreation”[Mesh] OR “Recreation”[tiab] OR “Recreations”[tiab] OR “Physical Fitness”[Mesh] OR “Physical Fitness”[tiab] OR “Cardiorespiratory Fitness”[Mesh] OR “Cardiorespiratory Fitness”[tiab] OR “Step count”[tiab] OR “Bicycling”[Mesh] OR “Bicycling”[tiab] OR “Biking”[tiab] OR “group exercise”[tiab] OR “group exercises”[tiab] OR “aquatic exercise”[tiab] OR “aquatic exercises”[tiab] OR “upper extremity exercise”[tiab] OR “upper extremity exercises”[tiab] OR “lower extremity exercise”[tiab] OR “lower extremity exercises”[tiab] OR “isometric exercise”[tiab] OR “isometric exercises”[tiab] OR “isokinetic exercise”[tiab] OR “isokinetic exercises”[tiab] OR “muscle strengthening”[tiab] OR “cycling”[tiab] OR “outdoor recreation”[tiab] OR “run”[tiab] OR “walk”[tiab] OR “jog”[tiab])  AND  (Randomized Controlled Trial[Publication Type] OR Controlled Clinical Trial[Publication Type] OR Clinical Trial[Publication Type] OR "Intervention"[tiab] OR "Health Promotion"[Mesh] OR “Health Promotion”[tiab] OR “Wellness Program"[tiab] OR "Wellness Programs"[tiab] OR "Obesity Management"[Mesh] OR “Obesity Management”[tiab] OR "Weight Reduction Programs"[Mesh] OR “Weight Reduction Programs”[tiab] OR “Weight Reduction Program”[tiab] OR “experimental studies”[tiab] OR “experimental study”[tiab] OR “intervention trial”[tiab] OR “intervention trials”[tiab])  **Limiters:** English[lang] |
| **Search strategy for PsychINFO** |
| (MAINSUBJECT.EXACT("Rural Environments") OR ti("Rural Environments") OR ab("Rural Environments") OR ti("Rural Environment") OR ab("Rural Environment") OR ti(“rural spatial distribution”) OR ab(“rural spatial distribution”) OR ti(“rural spatial distributions”) OR ab(“rural spatial distributions”) OR ti(“rural community”) OR ab(“rural community”) OR ti(“rural communities”) OR ab(“rural communities”) OR ti(“rural health service”) OR ab(“rural health service”) OR ti(“rural health services”) OR ab(“rural health services”) OR ti(“rural health center”) OR ab(“rural health center”) OR ti(“rural health centers”) OR ab(“rural health centers”) OR ti(“rural health”) OR ab(“rural health”) OR ti(“rural population”) OR ab(“rural population”) OR ti(“rural populations”) OR ab(“rural populations”) OR ti(“rural area”) OR ab(“rural area”) OR ti(“rural areas”) OR ab(“rural areas”) OR ti(“rural”) OR ab(“rural”) OR MAINSUBJECT.EXACT("Suburban Environments") OR ti("Suburban Environments") OR ab("Suburban Environments") OR ti("Suburban Environment") OR ab("Suburban Environment") OR ti(“suburban population”) OR ab(“suburban population”) OR ti(“suburban populations”) OR ab(“suburban populations”) OR ti(“suburban”) OR ab(“suburban”) OR ti(“suburban health”) OR ab(“suburban health”) OR ti(“suburban area”) OR ab(“suburban area”) OR ti(“suburban areas”) OR ab(“suburban areas”) OR ti(“suburbs”) OR ab(“suburbs”) OR ti(“suburbanization”) OR ab(“suburbanization”) OR ti(“nonmetropolitan population”) OR ab(“nonmetropolitan population”) OR ti(“nonmetropolitan populations”) OR ab(“nonmetropolitan populations”) OR ti(“farm”) OR ab(“farm”) OR ti(“farms”) OR ab(“farms”) OR ti(“farmland”) OR ab(“farmland”) OR ti(“farmlands”) OR ab(“farmlands”) OR ti(“vineyard”) OR ab(“vineyard”) OR ti(“vineyards”) OR ab(“vineyards”) OR ti(“micropolitan”) OR ab(“micropolitan”) OR ti(“nonmetropolitan”) OR ab(“nonmetropolitan”) OR ti(“nonmetro”) OR ab(“nonmetro”) OR ti(“nonmetropolitan area”) OR ab(“nonmetropolitan area”) OR ti(“nonmetropolitan areas”) OR ab(“nonmetropolitan areas”) OR MAINSUBJECT.EXACT("Agriculture") OR ti("Agriculture") OR ab("Agriculture") OR ti(“agricultural”) OR ab(“agricultural”) OR ti(“countryside”) OR ab(“countryside”) OR ti(“small town”) OR ab(“small town”) OR ti(“provincial”) OR ab(“provincial”) OR ti(“pastoral”) OR ab(“pastoral”))  AND  (MAINSUBJECT.EXACT("Exercise") OR ti("Exercise") OR ab("Exercise") OR ti("Exercises") OR ab("Exercises") OR MAINSUBJECT.EXACT("Aerobic Exercise") OR ti("Aerobic Exercise") OR ab("Aerobic Exercise") OR ti("Aerobic Exercises") OR ab("Aerobic Exercises") OR MAINSUBJECT.EXACT("Physical Activity") OR ti("Physical Activity") OR ab("Physical Activity") OR ti("Physical Activities") OR ab("Physical Activities") OR MAINSUBJECT.EXACT("Walking") OR ti("Walking") OR ab("Walking") OR MAINSUBJECT.EXACT("Running") OR ti("Running") OR ab("Running") OR MAINSUBJECT.EXACT("Weightlifting") OR ti("Weightlifting") OR ab("Weightlifting") OR ti("Weight Lifting") OR ab("Weight Lifting") OR ti("Weight Liftings") OR ab("Weight Liftings") OR MAINSUBJECT.EXACT("Swimming") OR ti("Swimming") OR ab("Swimming") OR MAINSUBJECT.EXACT("Recreation") OR ti("Recreation") OR ab("Recreation") OR ti("Recreations") OR ab("Recreations") OR MAINSUBJECT.EXACT("Physical Fitness") OR ti("Physical Fitness") OR ab("Physical Fitness") OR ti(“Physical Exercise”) OR ab(“Physical Exercise”) OR ti(“Physical Exercises”) OR ab(“Physical Exercises”) OR ti(“Acute Exercise”) OR ab(“Acute Exercise”) OR ti(“Acute Exercises”) OR ab(“Acute Exercises”) OR ti(“Exercise Training”) OR ab(“Exercise Training”) OR ti(“Exercise Trainings”) OR ab(“Exercise Trainings”) OR ti(“Ambulation”) OR ab(“Ambulation”) OR ti(“Human Physical Conditioning”) OR ab(“Human Physical Conditioning”) OR ti(“Human Physical Training”) OR ab(“Human Physical Training”) OR ti("Resistance Training") OR ab("Resistance Training") OR ti(“Circuit Based Exercise”) OR ab(“Circuit Based Exercise”) OR ti(“Circuit Based Exercises”) OR ab(“Circuit Based Exercises”) OR ti(“Circuit Training”) OR ab(“Circuit Training”) OR ti(“Circuit Trainings”) OR ab(“Circuit Trainings”) OR ti(“High Intensity Interval Training”) OR ab(“High Intensity Interval Training”) OR ti(“High Intensity Interval Trainings”) OR ab(“High Intensity Interval Trainings”) OR ti(“High Intensity Intermittent Exercise”) OR ab(“High Intensity Intermittent Exercise”) OR ti(“High Intensity Intermittent Exercises”) OR ab(“High Intensity Intermittent Exercises”) OR ti(“Sprint Interval Training”) OR ab(“Sprint Interval Training”) OR ti(“Sprint Interval Trainings”) OR ab(“Sprint Interval Trainings”) OR ti(“Plyometrics”) OR ab(“Plyometrics”) OR ti(“Plyometric Exercise”) OR ab(“Plyometric Exercise”) OR ti(“Plyometric Exercises”) OR ab(“Plyometric Exercises”) OR ti(“Plyometric Drill”) OR ab(“Plyometric Drill”) OR ti(“Plyometric Drills”) OR ab(“Plyometric Drills”) OR ti(“Plyometric Training”) OR ab(“Plyometric Training”) OR ti(“Plyometric Trainings”) OR ab(“Plyometric Trainings”) OR ti(“Stretch Shortening Exercise”) OR ab(“Stretch Shortening Exercise”) OR ti(“Stretch Shortening Exercises”) OR ab(“Stretch Shortening Exercises”) OR ti(“Stretch Shortening Cycle Exercise”) OR ab(“Stretch Shortening Cycle Exercise”) OR ti(“Stretch Shortening Cycle Exercises”) OR ab(“Stretch Shortening Cycle Exercises”) OR ti(“Stretch Shortening Drill”) OR ab(“Stretch Shortening Drill”) OR ti(“Stretch Shortening Drills”) OR ab(“Stretch Shortening Drills”) OR ti(“Aerobic Exercises") OR ab("Aerobic Exercises") OR ti(“Aerobic Exercise") OR ab("Aerobic Exercise") OR ti(“Strength Training”) OR ab (“Strength Training”) OR ti(“Weight Lifting Strengthening Program”) OR ab(“Weight Lifting Strengthening Program”) OR ti(“Weight Lifting Strengthening Programs”) OR ab(“Weight Lifting Strengthening Programs”) OR ti(“Weight Lifting Exercise Program”) OR ab(“Weight Lifting Exercise Program”) OR ti(“Weight Lifting Exercise Programs”) OR ab(“Weight Lifting Exercise Programs”) OR ti(“Weight Bearing Strengthening Program”) OR ab(“Weight Bearing Strengthening Program”) OR ti(“Weight Bearing Strengthening Programs”) OR ab(“Weight Bearing Strengthening Programs”) OR ti(“Weight Bearing Exercise Program”) OR ab(“Weight Bearing Exercise Program”) OR ti(“Weight Bearing Exercise Programs”) OR ab(“Weight Bearing Exercise Programs”) OR ti(“Step Count”) OR ab(“Step Count”) OR ti("Group Exercise") OR ab("Group Exercise") OR ti("Group Exercises") OR ab("Group Exercises") OR ti("Aquatic Exercise") OR ab("Aquatic Exercise") OR ti("Aquatic Exercises") OR ab("Aquatic Exercises") OR ti("Upper Extremity Exercises") OR ab("Upper Extremity Exercises”) OR ti("Upper Extremity Exercise”) OR ab("Upper Extremity Exercise”) OR ti("Lower Extremity Exercise") OR ab("Lower Extremity Exercise") OR ti("Lower Extremity Exercises") OR ab("Lower Extremity Exercises") OR ti("Isometric Exercises") OR ab("Isometric Exercises") OR ti("Isometric Exercise") OR ab("Isometric Exercise") OR ti("Isokinetic Exercises") OR ab("Isokinetic Exercises") OR ti("Isokinetic Exercise") OR ab("Isokinetic Exercise") OR ti("Cardiorespiratory Fitness") OR ab("Cardiorespiratory Fitness") OR ti("Cycling") OR ab("Cycling") OR ti("Muscle Strengthening") OR ab("Muscle Strengthening") OR ti("Bicycling") OR ab("Bicycling") OR ti("Biking") OR ab("Biking") OR ti(“Outdoor Recreation”) OR ab(“Outdoor Recreation”) OR ti("Run") OR ab(“Run") OR ti("Walk") OR ab("Walk") OR ti("Jog") OR ab("Jog"))  AND  (MAINSUBJECT.EXACT("Clinical Trials") OR ti("Clinical Trials") OR ab("Clinical Trials") OR MAINSUBJECT.EXACT("Health Promotion") OR ti("Health Promotion") OR ab("Health Promotion") OR ti("Randomized Controlled Trials") OR ab("Randomized Controlled Trials") OR ti("Weight Reduction Programs") OR ab("Weight Reduction Programs") OR ti("Experimental Studies") OR ab("Experimental Studies") OR ti("Intervention Trials") OR ab("Intervention Trials") OR ti("Intervention") OR ab("Intervention") OR ti("Wellness Program") OR ab("Wellness Program") OR ti("Obesity Management") OR ab("Obesity Management”))  **Limiters:** Language: English, Applied filters: Scholarly Journals |
| **Search strategy for CINAHL** |
| ((MH "Rural Population") OR TI "Rural Population" OR AB "Rural Population" OR TI “Rural Populations” OR AB “Rural Populations” OR (MH “Rural Health”) OR TI “Rural Health” OR AB “Rural Health” OR (MH “Rural Health Centers”) OR TI “Rural Health Centers” OR AB “Rural Health Centers” OR TI “Rural Health Center” OR AB “Rural Health Center” OR (MH "Rural Health Services") OR TI "Rural Health Services" OR AB "Rural Health Services" OR TI "Rural Health Service" OR AB "Rural Health Service" OR TI “Rural Area” OR AB “Rural Area” OR TI “Rural Areas” OR AB “Rural Areas” OR TI "Rural Community" OR AB "Rural Community" OR TI "Rural Communities" OR AB "Rural Communities" OR TI “Rural Environment” OR AB “Rural Environment” OR TI “Rural Environments” OR AB “Rural Environments” OR TI “Rural Spatial Distribution” OR AB “Rural Spatial Distribution” OR TI “Rural Spatial Distributions” OR AB “Rural Spatial Distributions” OR (MH "Suburban Population") OR TI "Suburban Population" OR AB "Suburban Population" OR TI "Suburban Populations" OR AB "Suburban Populations" OR TI “Nonmetropolitan Population” OR AB “Nonmetropolitan Population” OR TI “Nonmetropolitan Populations” OR AB “Nonmetropolitan Populations” OR TI “Suburbanization” OR AB “Suburbanization” OR TI “Farm” OR AB “Farm” OR TI “Farms” OR AB “Farms” OR TI “Farmland” OR AB “Farmland” OR TI “Farmlands” OR AB “Farmlands” OR TI “Vineyard” OR AB “Vineyard” OR TI “Vineyards” OR AB “Vineyards” OR TI “Rural” OR AB “Rural” OR TI “Suburban Environment” OR AB “Suburban Environment” OR TI “Suburban Environments” OR AB “Suburban Environments” OR TI “Suburban” OR AB “Suburban” OR TI “Suburban Health” OR AB “Suburban Health” OR TI “Suburban Areas” OR AB “Suburban Areas” OR TI “Suburban Area” OR AB “Suburban Area” OR TI “Suburbs” OR AB “Suburbs” OR TI “Micropolitan” OR AB “Micropolitan” OR TI “Nonmetropolitan” OR AB “Nonmetropolitan” OR TI “Nonmetro” OR AB “Nonmetro” OR TI “Nonmetropolitan Area” OR AB “Nonmetropolitan Area” OR TI “Nonmetropolitan Areas” OR AB “Nonmetropolitan Areas” OR TI “Countryside” OR AB “Countryside” OR TI “Small Town” OR AB “Small Town” OR TI “Provincial” OR AB “Provincial” OR TI “Pastoral” OR AB “Pastoral” OR (MH "Agriculture") OR TI "Agriculture" OR AB "Agriculture" OR TI “Agricultural” OR AB “Agricultural”)  AND  ((MH "Exercise") OR TI "Exercise" OR AB "Exercise" OR TI "Exercises" OR AB "Exercises" OR TI “Physical Activity” OR AB “Physical Activity” OR TI “Physical Activities” OR AB “Physical Activities” OR TI “Physical Exercise” OR AB “Physical Exercise” OR TI “Physical Exercises” OR AB “Physical Exercises” OR TI “Acute Exercise” OR AB “Acute Exercise” OR TI “Acute Exercises” OR AB “Acute Exercises” OR TI “Exercise Training” OR AB “Exercise Training” OR TI “Exercise Trainings” OR AB “Exercise Trainings” OR TI “Ambulation” OR AB “Ambulation” OR TI “Human Physical Conditioning” OR AB “Human Physical Conditioning” OR TI “Human Physical Training” OR AB “Human Physical Training” OR (MH "Resistance Training") OR TI “Resistance Training” OR AB “Resistance Training” OR TI “Circuit Based Exercise” OR AB “Circuit Based Exercise” OR TI “Circuit Based Exercises” OR AB “Circuit Based Exercises” OR TI “Circuit Training” OR AB “Circuit Training” OR TI “Circuit Trainings” OR AB “Circuit Trainings” OR TI “High Intensity Interval Training” OR AB “High Intensity Interval Training” OR TI “High Intensity Interval Trainings” OR AB “High Intensity Interval Trainings” OR TI “High Intensity Intermittent Exercise” OR AB “High Intensity Intermittent Exercise” OR TI “High Intensity Intermittent Exercises” OR AB “High Intensity Intermittent Exercises” OR TI “Sprint Interval Training” OR AB “Sprint Interval Training” OR TI “Sprint Interval Trainings” OR AB “Sprint Interval Trainings” OR (MH “Plyometrics”) OR TI “Plyometrics” OR AB “Plyometrics” OR TI “Plyometric Exercise” OR AB “Plyometric Exercise” OR TI “Plyometric Exercises” OR AB “Plyometric Exercises” OR TI “Plyometric Drill” OR AB “Plyometric Drill” OR TI “Plyometric Drills” OR AB “Plyometric Drills” OR TI “Plyometric Training” OR AB “Plyometric Training” OR TI “Plyometric Trainings” OR AB “Plyometric Trainings” OR TI “Stretch Shortening Exercise” OR AB “Stretch Shortening Exercise” OR TI “Stretch Shortening Exercises” OR AB “Stretch Shortening Exercises” OR TI “Stretch Shortening Cycle Exercise” OR AB “Stretch Shortening Cycle Exercise” OR TI “Stretch Shortening Cycle Exercises” OR AB “Stretch Shortening Cycle Exercises” OR TI “Stretch Shortening Drill” OR AB “Stretch Shortening Drill” OR TI “Stretch Shortening Drills” OR AB “Stretch Shortening Drills” OR TI "Resistance Training" OR AB "Resistance Training" OR (MH "Running") OR TI "Running" OR AB "Running" OR TI "Runnings" OR AB "Runnings" OR (MH "Walking") OR TI "Walking" OR AB "Walking" OR (MH "Aerobic Exercises") OR TI "Aerobic Exercises" OR AB "Aerobic Exercises" OR TI "Aerobic Exercise" OR AB "Aerobic Exercise" OR TI “Jogging" OR AB "Jogging" OR TI "Joggings" OR AB "Joggings OR TI “Strength Training” OR AB “Strength Training” OR TI “Weight Lifting Strengthening Program” OR AB “Weight Lifting Strengthening Program” OR TI “Weight Lifting Strengthening Programs” OR AB “Weight Lifting Strengthening Programs” OR TI “Weight Lifting Exercise Program” OR AB “Weight Lifting Exercise Program” OR TI “Weight Lifting Exercise Programs” OR AB “Weight Lifting Exercise Programs” OR TI “Weight Bearing Strengthening Program” OR AB “Weight Bearing Strengthening Program” OR TI “Weight Bearing Strengthening Programs” OR AB “Weight Bearing Strengthening Programs” OR TI “Weight Bearing Exercise Program” OR AB “Weight Bearing Exercise Program” OR TI “Weight Bearing Exercise Programs” OR AB “Weight Bearing Exercise Programs” OR TI “Step Count” OR AB “Step Count” OR (MH "Group Exercise") OR TI "Group Exercise" OR AB "Group Exercise" OR TI "Group Exercises" OR AB "Group Exercises" OR (MH "Aquatic Exercises") OR TI "Aquatic Exercises" OR AB "Aquatic Exercises" OR TI "Aquatic Exercise" OR AB "Aquatic Exercise" OR (MH "Upper Extremity Exercises") OR TI "Upper Extremity Exercises" OR AB "Upper Extremity Exercises" OR TI "Upper Extremity Exercise" OR AB "Upper Extremity Exercise" OR (MH "Lower Extremity Exercises") OR TI "Lower Extremity Exercises" OR AB "Lower Extremity Exercises" OR TI "Lower Extremity Exercise" OR AB "Lower Extremity Exercise" OR (MH "Isometric Exercises") OR TI "Isometric Exercises" OR AB "Isometric Exercises" OR TI "Isometric Exercise" OR AB "Isometric Exercise" OR (MH "Isokinetic Exercises") OR TI "Isokinetic Exercises" OR AB "Isokinetic Exercises" OR TI "Isokinetic Exercise" OR AB "Isokinetic Exercise" OR (MH "Swimming") OR TI "Swimming" OR AB "Swimming" OR (MH "Weight Lifting") OR TI "Weight Lifting" OR AB "Weight Lifting" OR TI "Weight Liftings" OR AB "Weight Liftings" OR (MH "Recreation") OR TI "Recreation" OR AB "Recreation" OR TI "Recreations" OR AB "Recreations" OR (MH "Physical Fitness") OR TI "Physical Fitness" OR AB "Physical Fitness" OR (MH "Cardiorespiratory Fitness") OR TI "Cardiorespiratory Fitness" OR AB "Cardiorespiratory Fitness" OR (MH "Cycling") OR TI "Cycling" OR AB "Cycling" OR (MH "Muscle Strengthening") OR TI "Muscle Strengthening" OR AB "Muscle Strengthening" OR TI "Bicycling" OR AB "Bicycling" OR TI "Biking" OR AB "Biking" OR TI “Outdoor Recreation” OR AB “Outdoor Recreation” OR TI "Run" OR AB "Run" OR TI "Walk" OR AB "Walk" OR TI "Jog" OR AB "Jog")  AND  ((MH "Randomized Controlled Trials") OR TI "Randomized Controlled Trials" OR AB "Randomized Controlled Trials" OR (MH "Clinical Trials") OR TI "Clinical Trials" OR AB "Clinical Trials" OR (MH "Health Promotion") OR TI "Health Promotion" OR AB "Health Promotion" OR (MH "Weight Reduction Programs") OR TI "Weight Reduction Programs" OR AB "Weight Reduction Programs" OR (MH "Experimental Studies") OR TI "Experimental Studies" OR AB "Experimental Studies" OR (MH "Intervention Trials") OR TI "Intervention Trials" OR AB "Intervention Trials" OR TI "Intervention" OR AB "Intervention" OR TI "Wellness Program" OR AB "Wellness Program" OR TI "Obesity Management" OR AB "Obesity Management”)  **Limiters:** English Language; Language: English |
| **Search strategy for PAIS** |
| (MAINSUBJECT.EXACT("Rural Population") OR ti("Rural Population") OR ab("Rural Population") OR MAINSUBJECT.EXACT("Rural Areas") OR ti("Rural Areas") OR ab("Rural Areas") OR MAINSUBJECT.EXACT("Rural Communities") OR ti("Rural Communities") OR ab("Rural Communities") OR MAINSUBJECT.EXACT("Suburbs") OR ti("Suburbs") OR ab("Suburbs") OR MAINSUBJECT.EXACT("Suburbanization") OR ti("Suburbanization") OR ab("Suburbanization") OR MAINSUBJECT.EXACT("Nonmetropolitan Areas") OR ti("Nonmetropolitan Areas") OR ab("Nonmetropolitan Areas") OR MAINSUBJECT.EXACT("Farms") OR ti("Farms") OR ab("Farms") OR ti("Rural Environments") OR ab("Rural Environments") OR ti(“rural spatial distribution”) OR ab(“rural spatial distribution”) OR ti(“rural community”) OR ab(“rural community”) OR ti(“rural communities”) OR ab(“rural communities”) OR ti(“rural health service”) OR ab(“rural health service”) OR ti(“rural health center”) OR ab(“rural health center”) OR ti(“rural health”) OR ab(“rural health”) OR ti(“rural”) OR ab(“rural”) OR ti("Suburban Environments") OR ab("Suburban Environments") OR ti(“suburban population”) OR ab(“suburban population”) OR ti(“suburban”) OR ab(“suburban”) OR ti(“nonmetropolitan population”) OR ab(“nonmetropolitan population”) OR ti(“farmland”) OR ab(“farmland”) OR ti(“vineyard”) or ab(“vineyard”) OR ti(“micropolitan”) OR ab(“micropolitan”) OR ti(“nonmetro”) OR ab(“nonmetro”) OR MAINSUBJECT.EXACT("Agriculture") OR ti("Agriculture") OR ab("Agriculture") OR ti(“agricultural”) OR ab(“agricultural”) OR ti(“countryside”) OR ab(“countryside”) OR ti(“small town”) OR ab(“small town”) OR ti(“provincial”) OR ab(“provincial”) OR ti(“pastoral”) OR ab(“pastoral”))  AND  (MAINSUBJECT.EXACT("Walking") OR ti("Walking") OR ab("Walking") OR MAINSUBJECT.EXACT("Recreation") OR ti("Recreation") OR ab("Recreation") OR MAINSUBJECT.EXACT("Outdoor Recreation") OR ti("Outdoor Recreation") OR ab("Outdoor Recreation") OR MAINSUBJECT.EXACT("Physical Fitness") OR ti("Physical Fitness") OR ab("Physical Fitness") OR MAINSUBJECT.EXACT("Cycling") OR ti("Cycling") OR ab("Cycling") OR ti("Exercise") OR ab("Exercise") OR ti("Aerobic Exercise") OR ab("Aerobic Exercise") OR ti("Physical Activity") OR ab("Physical Activity") OR ti("Physical Activities") OR ab("Physical Activities") OR ti("Running") OR ab("Running") OR ti("Weightlifting") OR ab("Weightlifting")ab OR ti("Swimming") OR ab("Swimming") OR ti(“Physical Exercise”) OR ab(“Physical Exercise”) OR ti(“Acute Exercise”) OR ab(“Acute Exercise”) OR ti(“Exercise Training”) OR ab(“Exercise Training”) OR ti(“Ambulation”) OR ab(“Ambulation”) OR ti(“Human Physical Conditioning”) OR ab(“Human Physical Conditioning”) OR ti(“Human Physical Training”) OR ab(“Human Physical Training”) OR ti("Resistance Training") OR ab("Resistance Training") OR ti(“Circuit Based Exercise”) OR ab(“Circuit Based Exercise”) OR ti(“Circuit Training”) OR ab(“Circuit Training”) OR ti(“High Intensity Interval Training”) OR ab(“High Intensity Interval Training”) OR ti(“High Intensity Intermittent Exercise”) OR ab(“High Intensity Intermittent Exercise”) OR ti(“Sprint Interval Training”) OR ab(“Sprint Interval Training”) OR ti(“Plyometrics”) OR ab(“Plyometrics”) OR ti(“Plyometric Exercise”) OR ab(“Plyometric Exercise”) OR ti(“Plyometric Drill”) OR ab(“Plyometric Drill”) OR ti(“Plyometric Training”) OR ab(“Plyometric Training”) OR ti(“Stretch Shortening Exercise”) OR ab(“Stretch Shortening Exercise”) OR ti(“Stretch Shortening Cycle Exercise”) OR ab(“Stretch Shortening Cycle Exercise”) OR ti(“Stretch Shortening Drill”) OR ab(“Stretch Shortening Drill”) OR ti(“Aerobic Exercises") OR ab("Aerobic Exercises") OR ti(“Weight Lifting Strengthening Program”) OR ab(“Weight Lifting Strengthening Program”) OR ti(“Weight Lifting Exercise Program”) OR ab(“Weight Lifting Exercise Program”) OR ti(“Weight Bearing Strengthening Program”) OR ab(“Weight Bearing Strengthening Program”) OR ti(“Weight Bearing Exercise Program”) OR ab(“Weight Bearing Exercise Program”) OR ti(“Step Count”) OR ab(“Step Count”) OR ti("Group Exercise") OR ab("Group Exercise") OR ti("Aquatic Exercise") OR ab("Aquatic Exercise") OR ti("Upper Extremity Exercises") OR ab("Upper Extremity Exercises”) OR ti("Lower Extremity Exercises") OR ab("Lower Extremity Exercises") OR ti("Isometric Exercises") OR ab("Isometric Exercises") OR ti("Isokinetic Exercises") OR ab("Isokinetic Exercises") OR ti("Cardiorespiratory Fitness") OR ab("Cardiorespiratory Fitness") OR ti("Muscle Strengthening") OR ab("Muscle Strengthening") OR ti("Bicycling") OR ab("Bicycling") OR ti("Biking") OR ab("Biking") OR ti("Run") OR ab(“Run") OR ti("Walk") OR ab("Walk") OR ti("Jog") OR ab("Jog"))  AND  (MAINSUBJECT.EXACT("Clinical Trials") OR ti("Clinical Trials") OR ab("Clinical Trials") OR MAINSUBJECT.EXACT("Intervention") OR ti("Intervention") OR ab("Intervention") OR ti("Health Promotion") OR ab("Health Promotion") OR ti("Randomized Controlled Trial") OR ab("Randomized Controlled Trial") OR ti("Weight Reduction Program") OR ab("Weight Reduction Program") OR ti("Experimental Study") OR ab("Experimental Study") OR ti("Intervention Trial") OR ab("Intervention Trial") OR ti("Wellness Program") OR ab("Wellness Program") OR ti("Obesity Management") OR ab("Obesity Management”))  **Limiters:** Additional limits - Language: English; Source type: Scholarly Journals |
| **Search strategy for Web of Science** |
| TS=("Rural Population" OR "Rural Populations" OR "Rural Spatial Distribution" OR “Rural Spatial Distributions” OR "Rural Communities" OR "Rural Community" OR "Rural Health Services" OR “Rural Health Service” OR "Rural Health Center" OR "Rural Health Centers" OR “Rural Health” OR "Suburban Population" OR "Suburban Populations" OR "Nonmetropolitan Population" OR "Nonmetropolitan Populations" OR "Suburbanization" OR "Farms" OR "Farm" OR "Farmland" OR "Farmlands" OR "Vineyard" OR "Vineyards" OR "rural" OR "micropolitan" OR "nonmetropolitan" OR "non-metropolitan" OR "nonmetro" OR "non-metro" OR "agricultural" OR "countryside" OR "small town" OR "provincial" OR "pastoral" OR “rural environment” OR “rural environments” OR “suburban environment” OR “suburban environments” OR “suburban” OR “agriculture” OR “rural area” OR “rural areas” OR “suburbs” OR “nonmetropolitan area” OR “nonmetropolitan areas”)  AND  TS=(“Exercise” OR “Exercises” OR “Physical Activity” OR “Physical Activities” OR “Physical Exercise” OR “Physical Exercises” OR “Acute Exercise” OR “Acute Exercises” OR “Isometric Exercises” OR “Isometric Exercise” OR “Aerobic Exercise” OR “Aerobic Exercises” OR “Exercise Training” OR “Exercise Trainings” OR “Running” OR “Runnings” OR “Jogging” OR “Joggings” OR “Walking” OR “Ambulation” OR “Human Physical Conditioning” OR “Human Physical Training” OR “Circuit-Based Exercise” OR “Circuit Based Exercise” OR “Circuit-Based Exercises” OR “Circuit Based Exercises” OR “Circuit Training” OR “Circuit Trainings” OR “High-Intensity Interval Training” OR “High Intensity Interval Training” OR “High Intensity Interval Trainings” OR “High-Intensity Interval Trainings” OR “High-Intensity Intermittent Exercise” OR “High-Intensity Intermittent Exercises” OR “High Intensity Intermittent Exercise” OR “High Intensity Intermittent Exercises” OR “Sprint Interval Training” OR “Sprint Interval Trainings” OR “Plyometric Exercise” OR “Plyometric Exercises” OR “Plyometric Drill” OR “Plyometric Drills” OR “Plyometric Training” OR “Plyometric Trainings” OR “Stretch-Shortening Exercise” OR “Stretch Shortening Exercise” OR “Stretch Shortening Exercises” OR “Stretch-Shortening Exercises” OR “Stretch-Shortening Cycle Exercise” OR “Stretch Shortening Cycle Exercise” OR “Stretch Shortening Cycle Exercises” OR “Stretch-Shortening Cycle Exercises” OR “Stretch-Shortening Drill” OR “Stretch Shortening Drill” OR “Stretch Shortening Drills” OR “Stretch-Shortening Drills” OR “Resistance Training” OR “Strength Training” OR “Weight-Lifting Strengthening Program” OR “Weight Lifting Strengthening Program” OR “Weight Lifting Strengthening Programs” OR “Weight-Lifting Strengthening Programs” OR “Weight-Lifting Exercise Program” OR “Weight Lifting Exercise Program” OR “Weight Lifting Exercise Programs” OR “Weight-Lifting Exercise Programs” OR “Weight-Bearing Strengthening Program” OR “Weight Bearing Strengthening Program” OR “Weight Bearing Strengthening Programs” OR “Weight-Bearing Strengthening Programs” OR “Weight-Bearing Exercise Program” OR “Weight Bearing Exercise Program” OR “Weight Bearing Exercise Programs” OR “Weight-Bearing Exercise Programs” OR “Swimming” OR “Weight Lifting” OR “Weight Liftings” OR “Recreation” OR “Recreations” OR “Physical Fitness” OR “Cardiorespiratory Fitness” OR “Step count” OR “Bicycling” OR “Biking” OR “group exercise” OR “group exercises” OR “aquatic exercise” OR “aquatic exercises” OR “upper extremity exercise” OR “upper extremity exercises” OR “lower extremity exercise” OR “lower extremity exercises” OR “isometric exercise” OR “isometric exercises” OR “isokinetic exercise” OR “isokinetic exercises” OR “muscle strengthening” OR “cycling” OR “outdoor recreation” OR “run” OR “walk” OR “jog”)  AND  TS=(“Randomized Controlled Trial” OR “Controlled Clinical Trial” OR “Clinical Trial” OR "Intervention” OR “Health Promotion” OR “Wellness Program" OR "Wellness Programs" OR “Obesity Management” OR “Weight Reduction Programs” OR “Weight Reduction Program” OR “experimental studies” OR “experimental study” OR “intervention trial” OR “intervention trials”)  **Limiters:** LANGUAGE: (English) AND DOCUMENT TYPES: (Article) |
