# Supplementary material for: Rural physical activity interventions in the United States: a systematic review and RE-AIM evaluation
Source: Int J Behav Nutr Phys Act. 2019 Dec 27;16:140. doi: 10.1186/s12966-019-0903-5 (PMC6935185; doi:10.1186/s12966-019-0903-5)
Supplement: Supplementary file 3 — Additional file 3. Characteristics of original studies included in review. Description: The citation details, sample characteristics, location/setting, rurality classification, and summary of findings of included articles. [file 12966_2019_903_MOESM3_ESM.docx]

**Additional File 3:** Characteristics of original studies included in review

| Author, year (reference) | Sample characteristics (sample size, target population) | Intervention (duration, description) | Location/  Setting | Classification of rurality | Summary of findings |
| --- | --- | --- | --- | --- | --- |
| Anson & Madras, 2016 (24) | *N*=80  Students, faculty and staff of a small university | 8 weeks  Website-based intervention, included incentivized pedometer-monitored program with step goals and daily feedback | Small rural university | Author self-stated | Subjects with high goals walked more than those with low goals (*p*<0.05). Reducing goals from a high to low level reduced walking behavior from 9700 steps to 9000 steps (*p*<0.05), and increasing goals from a low to high level increased walking behavior from 7900 steps to 9500 steps (*p*<0.05) |
| Befort et al., 2010 (25) | *N*=34  Overweight and obese women | 24 weeks  Group vs. individual phone counseling intervention, included weekly treatment sessions for 16 weeks followed by 4 biweekly sessions focused on goal-setting, self-monitoring, problem solving, stimulus control, and relapse prevention for diet and physical activity | Non-metropolitan Kansas counties | Population/Census based | Significant increases in physical activity from baseline to 6 months physical activity (+1235 ± 832 kcal/week or +196.5 ± 115.5 min/week); 71% of participants who completed the intervention met the 225 min/week physical activity goal at 6 months |
| Befort et al., 2012 (26) | *N*=31  Obese breast cancer survivors | 24 weeks  Group-based weight control intervention delivered through conference call technology, focused on goal-setting, self-monitoring, problem-solving, stimulus control and social support for diet and physical activity | Rural Kansas towns | Rural Urban Commuting Area (RUCA) codes | Significant increases in physical activity from baseline to 6 months physical activity (+1235 ± 832 kcal/week or +196.5 ± 115.5 min/week); 71% of participants who completed the intervention met the 225 min/week physical activity goal at 6 months |
| Benson et al., 2019 (27) | *N*=118  Adults with type 2 diabetes | 48 weeks (12 months)  Registered dietitian nutritionist-led telemedicine intervention program, included monthly telephone coaching with frequency tailored to patient preferences | Rural Minnesota communities | Author self-stated | No significant differences in self-reported physical activity between intervention and control groups |
| Campbell et al., 2002 (28-30) | *N*=538  Blue-collar female employees | 72 weeks (18 months)  Individualized computer-tailored health magazine and a “natural helpers” intervention that trained employees to diffuse information and provide support | Eastern North Carolina rural counties | Author self-stated | Significant improvements in frequency of strengthening and flexibility exercise in the intervention group at 18 months compared to the delayed group (*p*< 0.01); no statistically significant differences in frequency of aerobic activities or total METs between intervention and delayed groups at 18 months |
| Campbell et al., 2004 (31) | *N*=587  African American church members | 36 weeks  Lay health advisor-led intervention, included walking and exercise groups vs. mail-delivered tailored newsletters and four targeted videotapes | African American churches in rural eastern North Carolina counties | Author self-stated | Significant increase in self-reported recreational exercise (*p*=.04) in tailored video and print group compared to control. No significant difference in tailored print and video intervention or combined group compared to control |
| Campbell et al., 2012 (32-33) | *N*=485  Women | 24 weeks  Community-based interventions led by trained community leaders, included group sessions, discussion, goal-setting, 20-minute group activity sessions, and mailed monthly tailored health newsletters | Rural North Carolina counties | Author self-stated | Significant increases in self-reported physical activity (+51.8 ±19.2 minutes/week) in intervention group compared to control group (*p*=0.007) |
| Ely et al., 2008  (34-35) | *N*=107  Overweight and obese primary care patients | 24 weeks  Obesity chronic care model (CCM) program, included regular motivational interviewing (MI)-based telephone counseling, standardized weight loss materials, pedometers, and diet and physical activity diaries | Rural Kansas primary care practices | Population/Census based | No significant changes in self-reported moderate or vigorous physical activity from baseline to follow-up or between intervention and control groups |
| Fahs et al., 2013  (36) | *N*=117  Women | 56 weeks (14 months)  Community Intervention (CI), which included health fair and online list of accessible physical activity sites, vs. Stage Matched Nursing and Community Intervention (SMN+CI), which included Stages of Change (SOC)-based visits from registered nurses | Rural New York and Virginia counties | Rural-Urban Continuum Codes (RUCC) | No significant differences between groups for self-reported physical activity post-intervention |
| Farag et al, 2010  (37) | *N*=187  Public school system employees | 24 weeks  Worksite-based intervention including pedometers, informational booklets promoting walking, step goals, treadmills and walking posters placed in schools | Rural public school system in Southwestern Oklahoma | Population/Census based | No significant changes in self-reported physical activity from pre- to post-intervention |
| Fazzino et al., 2017  (38-40) | *N*=142  Post-menopausal female breast cancer survivors | 72 weeks (18 months)  Phone-based intervention, included a 6-month weight loss phase with weekly group phone sessions, DVDs, pedometers, and self-monitoring, followed by a 12-month weight loss maintenance phase with continued phone sessions vs. mailed newsletters | Rural areas of the Midwestern United States | Author self-stated | Significant increases in accelerometer-measured median MVPA bouted min from baseline to 6 months (18.4 vs 65.3, *p*=.001, 350% increase), significant decreases from 6 to 18 months (65.3 vs 38.1, *p*=.01; 42% decrease). MVPA at 18 months remained significantly higher than baseline (18.4 vs 38.1, *p*=.001; 210% increase)  Significant increases in self-reported MVPA min/week from baseline to 6 months (0 vs 227.5, p=.001; 227% increase), significant decreases from 6 to 18 months (227.5 vs 150.0; 34% decrease). Self-reported MVPA at 18 months remained significantly higher than baseline (150% increase). |
| Folta et al., 2009  (41) | *N*=87  Sedentary, overweight, midlife or older women | 12 weeks  Physical activity (dancing or walking) classes, self-monitoring and goal setting | Rural Kansas and Arkansas counties | Rural-Urban Continuum Codes (RUCC) | Significant increase in number of steps/day in intervention groups compared with control groups (1637 steps/day; 95% CI=712, 2562) |
| Gore et al., 2019  (42) | *N*=204  Medically-underserved community residents | 48 weeks (12 months)  Mobile health intervention using motivational SMS messages integrated into a community CVD risk reduction program. involving community health workers | Rural Colorado communities | Author self-stated | No significant difference in the pro-portion of participants who self-reported an increase in exercise in the SMS intervention group compared to control group |
| Greaney et al., 2017  (43-44) | *N*=121  Socio-economically disadvantaged overweight and obese Black women | 48 weeks (12 months)  Tailored behavior change goals, skills training materials, weekly interactive voice response (IVR) telephone calls for self-monitoring, monthly telephone coaching from a registered dietitian, and a YMCA membership | Rural North Carolina community health centers | Author self-stated | No significant change in accelerometer-measured MVPA over the intervention period  No significant difference in changes in accelerometer-measured MVPA by group assignment over the intervention period |
| Griffin et al., 2014  (45) | *N*=104  Low income overweight and obese women | 12 weeks  Mobile health intervention using text messages and weekly electronic newsletters, included health tips, reminders, and goal-setting prompts | Rural Alabama counties | Author self-stated | Significant improvement in self-reported physical activity from pre- to post-assessment (*p*<0.05) |
| Hageman et al., 2014  (46) | *N*=289  Midlife and older women with pre-hypertension | 48 weeks (12 months)  Web-based vs. print-mailed intervention, included individual telephone goal-setting counseling, self-monitoring, exercise videos, and newsletters with content-specific information and tailored behavioral messaging | Rural central Nebraska areas | Rural Urban Commuting Area (RUCA) codes | Significant increase in cardiorespiratory fitness, defined as estimated VO_2_ max, (*p*=.037) in web-based intervention group compared to control  No significant changes in self-reported physical activity from baseline in web-based or print-mailed intervention groups compared to control |
| Hu et al., 2014  (47) | *N*=36  Patients with diabetes and 37 family members | 8 weeks  Family-based intervention, included family and patient group sessions, seminar discussions, educational flipcharts and games, video-tapes, demonstrations, and self-monitoring | Rural central North Carolina | Author self-stated | No significant increases in self-reported physical activity from pre- to post-intervention in patients with diabetes or in family members |
| Keyserling et al., 2016  (48-49) | Phase I: *N*=251  Phase II: *N*=138  Phase III: *N*=27  Residents of Lenoir County, North Carolina | 96 weeks (24 months)  Phase I (months 1-6): Individually-tailored intervention promoting a Mediterranean-style diet and increased walking; Phase II (months 7-12): Option of weight loss intervention offered in two formats (16 weekly group sessions or 5 group sessions and 10 phone calls) or a lifestyle maintenance intervention; Phase III (months 13-24): Weight loss maintenance intervention for those losing ≥8 lb or a lifestyle maintenance intervention | Lenoir County, North Carolina | Author self-stated | Phase I: Increase in walking time of 64 min/week (*p*≤0.01) and increase in total physical activity time of 97 min/week (*p*≤0.01) from baseline to 6 months  Phase II: Increase in walking time of 71 min/week (*p*≤0.001) and increase in total physical activity time of 83 min/week (*p*≤0.01) from baseline to 12 months  Phase III: No significant increase in walking time or total physical activity time from baseline to 24 months |
| Kim et al., 2008  (50) | *N*=61  African American church members | 8 weeks  Faith-based lay advisor-led intervention, included weekly learning modules and small group meetings, exercise tapes, Bible studies about health, and prayer | Rural African American faith communities in North Carolina | Author self-stated | Significant increase in self-reported mean recreational physical activity over time in intervention group (*p*<.05)  Significantly greater self-reported mean recreational physical activity from baseline to follow-up (*p*=.01) in intervention participants compared to control participants |
| Lilly et al., 2014  (51) | *N*=81  Three underserved communities (Hispanic/non-Hispanic white women, African American women, Appalachian residents) | 8 weeks  Community-based intervention included in-person group sessions led by trained interventionists where participants identified barriers and learned problem-solving skills relevant to improving lifestyle behaviors known to affect CVD risk in the context of stressful life circumstances | Site 1: worksites in rural southern Colorado, Site 2: African  American church in semirural central North Carolina, Site 3: Appalachian community center | Author self-stated | No significant difference in self-reported physical activity from baseline to follow-up in total study group  No significant increases in self-reported physical activity from baseline to follow-up within sites |
| Marigliano et al., 2016  (52) | *N*=62  Women | 10 weeks  Trackable pedometer-based walking program, included an in-person talk about heart health, an individualized review of participants’ pedometer data with the principle investigator, and an incentivized raffle challenge midway through the study | Rural New York community | U.S. Office of Management and Budget (OMB) | Significant improvement in 6-minute walk test post-intervention (*p*<.05)  Significant increase in aerobic steps from midway through the end of the study (*p*<.05) |
| Parker et al., 2010  (53) | *N*=28  African American women | 10 weeks  Church-based spiritually-based vs nonspiritually-based interventions, included educational group sessions led by county extension educator, discussions with health providers, and biblical scriptures incorporated in spiritually-based sessions | Churches in rural South Carolina | Author self-stated | Significant increase in self-reported physical activity in spiritually-based intervention from pre- to post-intervention (*p*<.01)  No significant changes in self-reported physical activity in nonspiritually-based intervention from pre- to post-intervention. |
| Scarini et al., 2014  (54) | *N*=565  Middle-aged African  American women | 5 weeks  Community-based lifestyle intervention, included four group sessions and one individual session addressing knowledge and skills relating to physical activity and healthy eating choices | Rural counties in Alabama Black Belt | Alabama Rural Health Association | Significantly higher self-reported physical activity engagement in intervention arm (24% increase) compared to control arm (3% increase) at 12 months (*p*<.0001) |
| Spurrier et al., 2018  (55) | *N*=15  Overweight and obese adults | 12 weeks  Health promotion program, included in-person educational modules focusing on physical activity and nutrition, increasing knowledge of healthy activity and nutrition, and improving weight and BMI | Rural West Virginia community | Author self-stated | Significant increase in self-reported physical activity from pre- to post-intervention (*p*=.04) |
| Thomson et al., 2016  (56-57) | *N*=82  Mothers and their infants | 72 weeks (18 months)  Maternal, Infant, and Early Childhood Home Visiting (MIECHV) program delivered at home, intervention included culturally tailored physical activity and nutrition components specifically designed for the gestational and postnatal periods | Rural Mississippi Delta region | Author self-stated | No significant differences in self-reported physical activity by intervention arm or by gestational visit |
| Tussing-Humphreys et al., 2013  (58) | *N*=403  African American adults | 24 weeks  Church-based lifestyle intervention, included monthly educational sessions, newsletters, and a pedometer-based walking program | Rural Lower Mississippi Delta (LMD)  region of Mississippi | Author self-stated | Significant increases in self-reported aerobic physical activity (*p*=.02) and strength/flexibility physical activity (*p*=.03) in the intervention but not the control group |
| Warren et al., 2010  (59-60) | *N*=188  Female employees | 10 weeks  Worksite-based intervention, included individualized daily goals and email feedback, walking groups, weekly messages about group walking achievement, visible pedometers, management support, posted walking maps and walking circuits | Rural Upstate New York worksites | Author self-stated | Significant mean increase in daily steps for all intervention weeks, from a baseline weekly mean step value of 40,870±1,305 (5,839 steps/day) to 51,397± 524 steps/week (7,342 steps/day) (*p*<.01) |
| Wilcox et al., 2013  (61-62) | *N*=1257  Members of African Methodist Episcopal (AME) churches | 60 weeks (15 months)  Faith-based intervention, included Faith, Activity, and Nutrition (FAN) Program bulletin boards and committees, and physical activity and healthy eating church activities | Churches in rural South Carolina | Author self-stated | Significant increase in self-reported leisure-time MVPA in intervention churches (*p*=0.02), and decrease in control churches |
| Zoellner et al., 2013  (63) | *N*=58  Residents of Caswell County, North Carolina | 15 weeks  Community-based intervention included two weekly group fitness classes, with vs. without weekly nutrition and physical activity education sessions | Rural county in North Carolina | Rural-Urban Continuum Codes (RUCC) | Significant increase in self-reported weekly minutes of moderate physical activity (*p*<.003) in both groups (with vs. without educational sessions) |
